# Do high soil temperatures on Namibian fairy circle discs explain the absence of vegetation?

**DOI:** 10.1371/journal.pone.0217153

**Published:** 2019-05-20

**Authors:** Kelly Vlieghe, Mike Picker

**Affiliations:** Department of Biological Sciences, University of Cape Town, Cape Town, South Africa; Chinese Academy of Forestry, CHINA

## Abstract

Evenly-dispersed enigmatic bare discs known as ‘fairy circles’ occur within grasslands of the pro-Namib Desert. In spite of their conspicuous appearance, their nature and origin is still debated. The possible inhibitory effects of high surface and sub-surface soil temperatures on grass germination and seedling development on fairy circles have not yet been investigated. We measured maximum, mean daily (24 hour) and mean daytime (sunrise to sunset) temperatures of fairy circles and matrices in the NamibRand Nature Reserve (southwest Namibia). Optimum germination and growing temperatures, and thermal maxima of *Stipagrostis ciliata*, a grass commonly associated with fairy circle grasslands, were determined experimentally in growth trials. Seeds and seedlings were exposed to temperatures of 35 °C, 37 °C, 41 °C, 44 °C and 47 °C for 10 days. The optimum growth temperature range of *S*. *ciliata* seedlings was determined to be 35 °C to 37 °C, with depressed growth above 47 °C. Seed germination was also depressed at 47 °C, and optimal germination occurred between 35 °C to 37 °C. Circle soils were consistently 2 °C cooler than matrix soils at both surface and 15 cm depths, and though the soil surface achieved daily temperatures of 45 °C and 47 °C for the circle and matrix respectively, mean daily temperatures at 15 cm depth were 36 °C and 38 °C respectively, coinciding with the optimum germination and growing temperature of *S*. *ciliata*. Circle soil temperature is thus unlikely to limit *S*. *ciliata* germination and seedling growth and contribute to the maintenance of a bare disc, as both thermal conditions and the presence of higher soil moisture on circles provide a more favourable growing environment than the matrix.

## Introduction

Species-poor grasslands along the pro-Namib Desert support evenly spaced, oval bare patches known as fairy circles (FCs). These 2–12 m diameter circles often display a peripheral ring of luxuriant grass [1,2] and demonstrate a distinctly high degree of hexagonal overdispersion [3,4]. Their origin is contested by two main theories (see [2,5] for a review of other theories of origin). The plant competition hypothesis [6,7] states that FCs are the result of an uneven distribution of water and nutrients towards more competitive grass patches, resulting in the death of neighbouring grasses and the generation of evenly-spaced bare patches. The support is largely model-based, including i) patch similarities to partial differential equation models based on vegetation patterns elsewhere in the world [7] and ii) the use of linear models to compare FC properties with local edaphic properties, and the use of boosted regression tree models to compare circle occurrence against regional climate and vegetation characteristics [6]. In contrast, the Sand termite hypothesis uses ground-truthed ecological data to demonstrate that the destructive foraging of the subterranean Sand termite (*Psammotermes allocerus* Silvestri) on the roots and culms of grasses above their buried nest, generates a circular bare patch as a result of central-based foraging [5,8]. The theory is supported by spatial associations of *P*. *allocerus* with FCs [5], incremental fluctuations in termite numbers with FC growth stages [8], the presence of the termite nests below circles [5,8], and herbivory trials which demonstrated that Sand termites kill living grasses through foraging on their roots [8]. Recently it has been proposed that these two competing theories are not mutually exclusive, and that FC’s and similar regularly spaced patterns may be formed by an interplay of both social insect self-organisation and vegetative scale-dependent feedbacks [9].

A number of edaphic factors on FC’s have been investigated in an attempt to explain their lack of grass cover. These include soil particle size [6,10], compaction [10], soil moisture content and infiltration [1,5,6,10,11], nutrients [2,6,10], pH [2,6], allellopathic chemicals [2,12,13], microbial activity [14], hydrocarbons [15] and radiation [2]. However, the influence of these factors on the growth of grasses has not investigated. A factor recently revisited to explain grass death on FC is the presence of allelopathic chemicals in circle soils. Meyer et al. [13] found traces of the triterpenoid euphol in soil samples from FCs and within tissues of *Euphorbia gummifera*: they were only able to detect trace amounts of the same chemical in soils outside of FCs not associated with *E*. *gummifera*. Though Meyer et al. [13] did not propose a definitive mechanism for FC formation, they proposed that euphol may negatively influence microbial communities in FC soils, impacting plant growth. Cramer and Barger [6] compared soil macronutrients, moisture, pH, electrical resistance and particle size of FC’s with those of the matrix, and although organic carbon, nitrogen, potassium and phosphorus levels were significantly lower on FCs, soil moisture content was 2-3-fold higher than the matrix. This observation of elevated soil moisture content on FCs is well documented [1,5,8,11] and has been accredited to a reduced rate of transpiration and larger soil pore sizes on FCs, resulting in higher water infiltration rates and reduced evaporative loss [5].

One factor that could impact grass growth on FCs is soil temperature. Temperature is the second most important environmental factor affecting seed germination after water and can influence the rate of germination and either lift or induce dormancy [16,17]. The breaking of primary dormancy in desert-adapted plants, induction of secondary dormancy or optimal germination conditions often fall within a specific temperature regime for that species, related to typical temperature fluctuations in the growing season [18]. In arid environments high temperatures can indirectly influence vegetation growth due to the close relationship between temperature and evaporation [19]. Plants adapted to hot desert environments have increased tolerance to high temperature extremes and have rapid growth during good rainfall periods when the effect of evaporation is lessened [19,20]. Sustained exposure to increased temperature in desert environments, however, can result in selective mortality, leading to changes in community composition [21]. Long-term climate change monitoring in the Sonoran Desert revealed that a decrease in cover of various woody leguminous species, could be attributed to direct heat damage to seedling roots, increased evapotranspiration and lowered resilience to low precipitation years [21].

At a smaller scale, shading effects provided by patches of vegetation lower the soil water evaporation rate, which facilitates growth in vegetated areas [22,23]. Alternatively the higher degree of radiation and resultant evaporation on patches with little or no vegetation cover may, along with other factors, inhibit the establishment of vegetation on these bare patches [24–26]. Here we investigate the link between upper soil temperatures of FC’s and matrix, and grass recruitment and growth. Soil temperature in the Namib Desert have been reported to reach exceptionally high levels (80 °C [27]), and an additional increase in soil temperature on FCs could negatively impact grass seed dormancy, germination, and growth on the bare disc, resulting in the long term maintenance of the FC’s bare surface. In order to link soil temperatures with grass germination and growth we also investigated the thermal maxima and optimal temperatures for seed germination and seedling growth and survival. We hypothesize that high soil temperatures may play a role in the continued maintenance of the bare disc of fairy circles by depressing seedling recruitment and growth.

## Methods

### Study site

Soil temperatures were taken from FC’s and adjacent matrixes at NamibRand Nature Reserve, south-western Namibia (25°00’44.8”S, 16°00’17.16”E). NamibRand has an arid climate with mild winters and hot summers. Rainfall mostly occurs in the summer months (December to May) with 70–80 mm of rainfall received annually [28]. Mean monthly temperatures range from ~11 °C in winter to ~25 °C in summer. Soils in the area are nutrient impoverished [6] and can be classified as pre-dominantly silt sand, based on the sediment textural classification model proposed by [29]. The reserve consists of both southern Namib sand seas and semi-desert vegetation types [30]. FCs occur in a flat grassland plain comprised of Namib sand drift interspersed with mountains and inselbergs, as well as in grassy valleys between longitudinal dunes to the west. Compositionally the grasslands are dominated by *Stipagrostis obtusa* (Delile) Nees, interspersed with patches of *Stipagrostis ciliata* (Desf.) and *Stipagrostis uniplumis* (Licht.) [3,6]. These *Stipagrostis* species are drought-resistant perennial grasses widespread in the dry south western parts of Namibia, and are often found together in abundance in dry, sandy habitats [31]. *S*. *ciliata* is highly adapted to environments with limited water availability and high temperatures [32]. Within NamibRand Nature Reserve both grass species are associated with the peripheral band of FCs; with *S*. *obtusa* being most prevalent in the early stages of a FC’s development, and subsequently being replaced by *S*. *ciliata* once the FC matures, possibly in response to elevated moisture levels within the soil of the FC bare disc [8].

### Soil temperature on and off FCs

Soil temperature was measured on 20 FCs and adjacent matrices using Dallas Thermochron DS1921G-F5 temperature data loggers. Measurements were taken over a 2–3 day period in the austral summer of February 2013, with a 10 minute recording interval. This period coincides with the summer rainfall period high soil surface temperatures and *Stipagrostis* germination [33]. The data loggers were buried in the centre of the bare circle disc, and approximately 4 m away from the circle in the matrix. For each FC and its matrix replicate, loggers were placed within a sealed Ziploc bag emptied of air and buried at both 15 cm and 1 cm below the surface. At the end of each 2–3 day logging period, the loggers were removed and the data downloaded digitally using Netgen SoftButton [34] data logging software. The mean temperature for each 10 min time interval over the 2–3 day period was determined for each sample, and daily (24 hr) mean, daytime (sunrise to sunset) mean, night-time mean (sunset to sunrise), minimum and maximum temperatures for the circle and matrix on the surface and at 15 cm depth calculated.

### Effect of high temperature on *Stipagrostis* seed germination and growth in laboratory trials

To investigate the potential adverse effects of high temperatures on *Stipagrostis* germination and seedling growth and to determine the thermal maxima, germination and seedling growth trials were conducted at five different temperatures using *S*. *ciliata* seed obtained from Renu-Karoo seed supplies, Prince Albert, South Africa. *S*. *ciliata* seed was collected from Prince Albert (Western Cape Province) in March 2011 and stored within their awns in dry, room temperature conditions, until the germination trials were conducted from September 2011 until December 2011. Seeds were grown in 8 cm deep plastic pots using sandy soil that was kept moist. To maintain constant soil temperatures, the drainage holes on the bottom of the pots were carefully sealed, and the pots immersed in five thermostatically-controlled water baths, each maintaining a different soil temperature. These were situated indoors in an area experiencing normal summer sunlight exposure for the duration of the experiment. *Stipagrostis* seeds are known to have low viability under laboratory conditions [35], thus two pilot experiments were conducted to inform the optimum experimental temperature range for growth, best seed thermal stratification technique (chilling), and effect of pre-wetting on germination (see S1 Text for details). According to previous stratification experiments for *S*. *ciliata* [35], a cold stratification was found to be most effective. The pilot experiments revealed 1) that the viability of *S*. *obtusa* seeds was low compared to that of *S*. *ciliata* 2) that stratification at 5 °C for three days [35] did not improve seed germination 3) that soaking the seeds for three days before planting and keeping them moist throughout the experiment increased germination. On the basis of the pilot studies, temperature treatments of 35 °C, 37 °C, 41 °C, 44 °C and 47 °C were chosen for both experiments (germination success, growth rate) as pilot studies indicated that the optimal growing temperature was higher than 30°C and the maximum temperature threshold for seedling survival was around 50 °C (no germination occurred at or above this temperature).

For the first seed heat tolerance trial, 16 replicate pots with 25 *S*. *ciliata* seeds in each were exposed to soil temperatures of 35 °C, 37 °C, 41 °C, 44 °C and 47 °C for nine days, for six hours each day. The seeds were soaked in water for three hours before the experiment but not stratified. The number of germinations was recorded each day, and at the end of the experiment the seedlings were carefully removed and the number of leaves, leaf and root length and number of secondary roots were recorded.

For the second experiment, S. *ciliata* seeds were allowed to germinate and grow naturally in sand-filled pots exposed to natural summer sunlight for two weeks. The seedlings were then transplanted into 16 replicate pots, with one seedling allocated to each replicate. The seedlings were allowed to establish for five days before being exposed for 10 days to soil temperatures of 35 °C, 37 °C, 41 °C, 44 °C and 47 °C for six hours each day. Leaf number and length, as well as root number and length, were recorded prior to transplantation and again at the end of the experiment.

### Statistical analyses and data availability

All statistical analyses were done using STATISTICA version 10 [36]. Parametric tests were used where all assumptions for each statistical test were met, otherwise non-parametric equivalents were used. For the soil temperature comparison of matrix and FC soils, Mann-Whitney U statistical tests of unpaired datasets were used. A Kruskal Wallis test between multiple datasets was used to compare temperature differences between surface and 15 cm soil depth. A generalised linear model was used to investigate the effect of temperature on growth responses in transplanted *S*. *ciliata* seedlings, and a Kruskal Wallis test was done to test for significant differences in seed germination between the five temperature treatments. Permutated MANOVA tests were done to test for significance in germinated seedling root number, root length and leaf length between the temperature treatments, as well as for changes in transplanted seedling root length, leaf length, unchlorosed leaf and chlorosed leaf number between the five temperature treatments. Raw data is provided as supplementary information (S1 Dataset).

## Results

### FC and matrix soil temperatures

**Fig 1:**
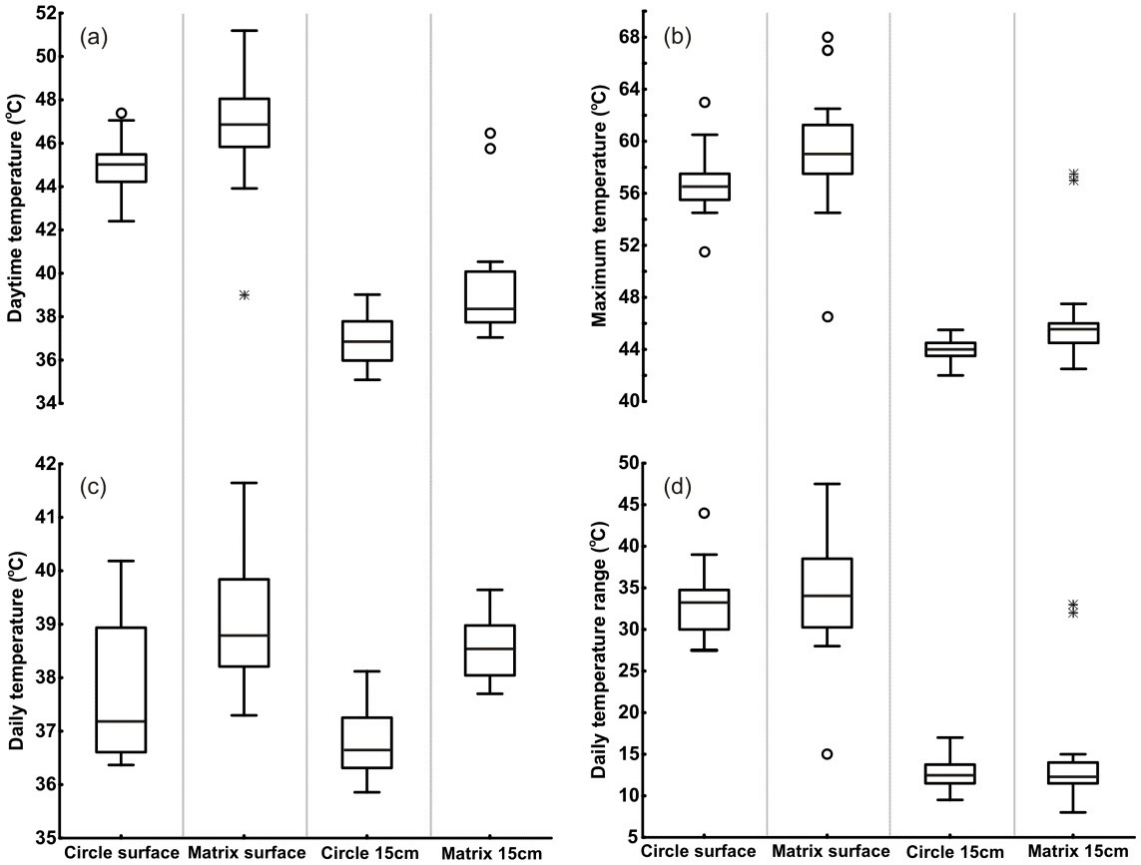
Box and whisker plots of (a) mean daytime temperature, (b) maximum temperature, (c) mean daily temperature and (d) mean daily temperature range of soils on the surface and at 15cm depth on FCs and the matrix (n = 20). Outliers = o, extremes = *.

The highest soil surface temperature was recorded in the matrix (63 °C), with matrix soils having higher daily, daytime, night time and maximum soil surface temperatures (mean ± SD): 39.1 °C ± 1.23 °C, 46.8 °C ± 2.60 °C, 30.7 °C ± 2.60 °C, 59.5 °C ± 4.72 °C respectively) than those of FCs (37.7 °C ± 1.24 °C, 45.0 °C ± 1.23 °C, 29.7 °C ± 1.23 °C, 56.7 °C ± 2.42 °C respectively) (Fig 1) (Mann-Whitney U, U_20,20_ = 83.5, *P* = 0.002 for daily mean, U_20,20_ = 73.0, *P* = 0.001 for daytime mean and U_20,20_ = 85.0, *P* = 0.002 for maximum temperature). The degree of surface temperature fluctuation did not differ between the sites (U_20,20_ = 170.5, *P* = 0.43) (Fig 1). Daytime temperatures, maximum temperatures and temperature range were lower at 15 cm depth compared to the surface for both FCs and the matrix (Kruskal Wallis, H_3,80_ = 60.65, *P* = 0.000 for daytime mean, H_3,80_ = 60.87, *P* = 0.000 for maximum and H_3,80_ = 54.08, *P* = 0.000 for temperature range).

Similar patterns to those of the surface soils were observed for daily temperatures (36.8 °C ± 0.68 °C for FCs, 38.6 °C ± 0.63 °C for matrix), daytime temperatures (36.9 °C ± 1.10 °C for FCs, 39.3 °C ± 2.56 °C for matrix), night time temperatures (36.8 °C ± 1.10 °C for FCs, 37.8 °C ± 2.56 °C for matrix) and maximum temperatures (43.9 °C ± 0.96 °C for FCs, 46.4 °C ± 3.91 °C for matrix) 15 cm soil depth (Mann-Whitney U, U_20,20_ = 15.0, *P* < 0.001 for daily mean, U_20,20_ = 54.0, *P* < 0.001 for daytime mean, U_20,20_ = 71.5, *P* = 0.001 for maximum and U_20,20_ = 182.5, *P* = 0.460 for temperature range) (Fig 1). The maximum recorded temperature at 15 cm depth was 48 °C in the matrix.

### Seed germination

**Fig 2:**
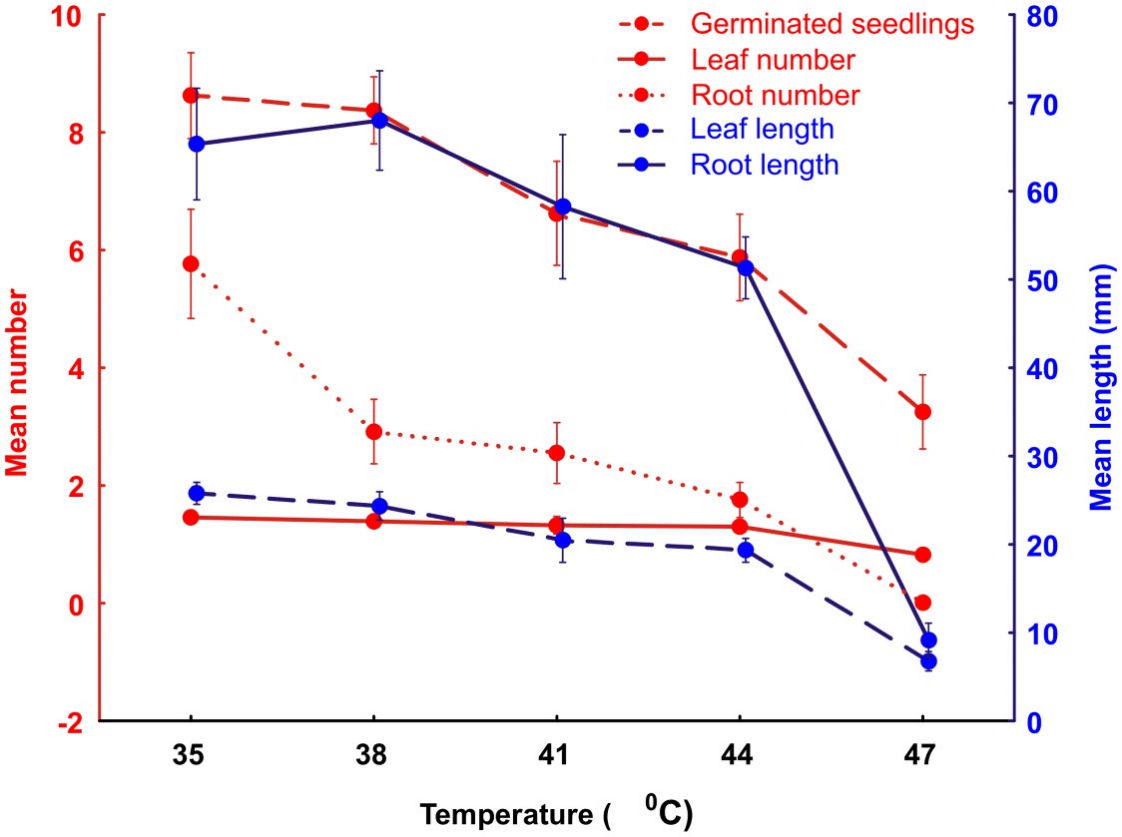
The mean number of germinated *S*. *ciliata* seeds, seedling leaf number, root number, leaf length and root length at different temperatures after nine days (n = 16 replicates). Error bars = standard errors.

The number of *S*. *ciliata* seeds germinated in the laboratory decreased significantly with increasing temperature along the gradient of 35 °C, 37 °C, 41 °C, 44 °C and 47 °C (R^2^ = - 0.947, F = 53.80, *P* = 0.005, n = 16), with the greatest germination occurring at the lowest experimental temperature (35 °C) (Kruskal Wallis, H_4,80_ = 26.78, *P* = 0.000, n = 16) (Fig 2).

At the end of the thermal trials, the number of roots as well as leaf and root length of the germinated seedlings were significantly lower or smaller with increasing temperature (Permutated MANOVA, F = 14.16_4, 80_, *P* = 0.0001, n = 16) (Fig 2). Plant growth performance factors (root and shoot length) were most severely impacted in the 44 °C exposure trial.

### Seedling growth

**Fig 3:**
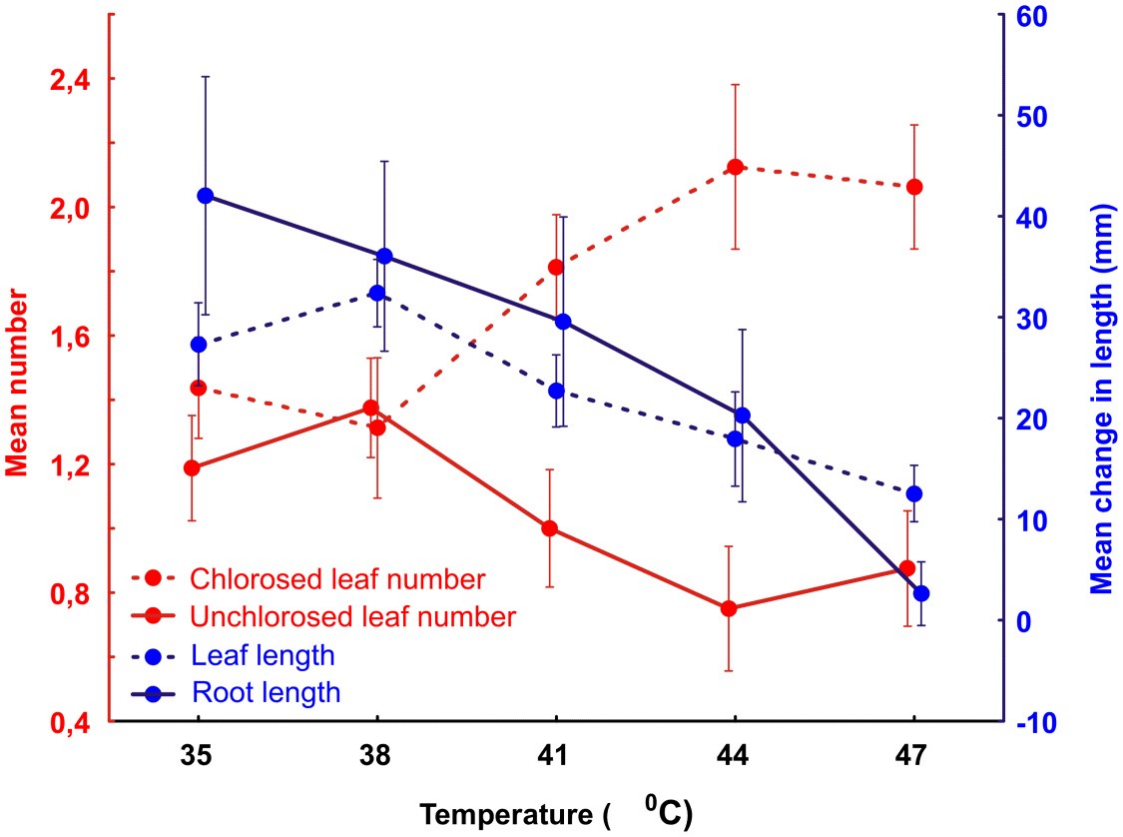
The mean number of unchlorosed and chlorosed leaves, and the change in root length and leaf length of transplanted *S*. *ciliata* seedlings after 10 days exposure to different temperatures (n = 16 replicates). Error bars = standard error.

**Fig 4:**
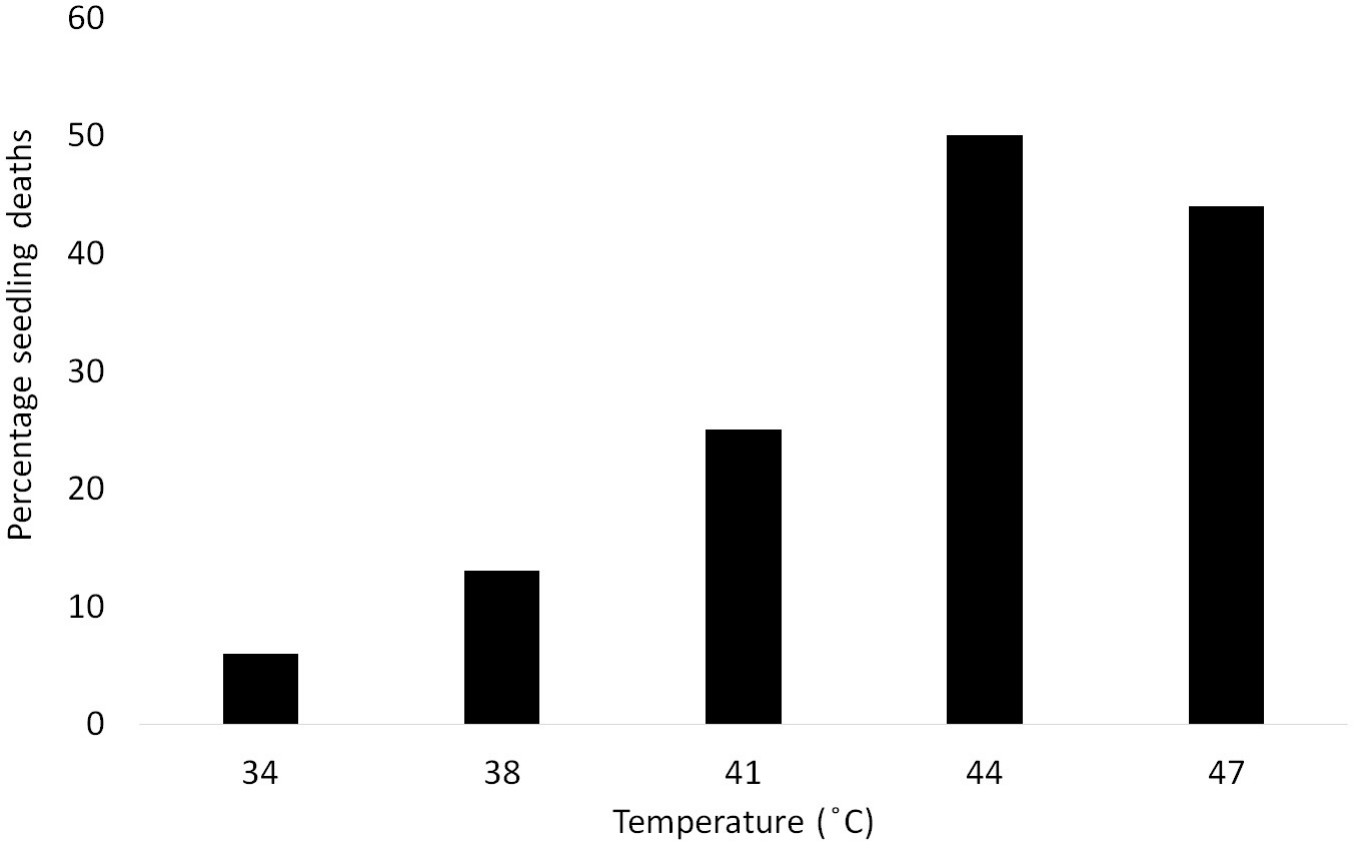
Percentage of transplanted *S*. *ciliata* seedling deaths after 10 days exposure to different temperatures (n = 16 replicates).

In the second thermal tolerance trial subjecting two-week old *S*. *ciliata* seedlings to the same temperature range for 10 days, seedling condition decreased significantly with increasing temperature, with root and leaf length as well as unchlorosed leaf number decreasing with temperature, and the number of leaves becoming chlorosed (dying) increasing (Permutated MANOVA, F = 3.075, *P* = 0.006) (Fig 3). The optimal temperature for growth, as evidenced by maximum leaf length and number was 37 °C, although root length decreased at all temperatures below 35 °C. The number of seedling deaths increased with temperature (χ^2^ = 8.45, *P* = 0.076) (Fig 4).

## Discussion

The striking feature of Namibian fairy circles is their persistent near absence of any grass cover, especially when compared to the densely vegetated matrix, which is dominated by species of perennial *Stipagrostis* grasses [6]. The natural cycle of recruitment is pulsed by the timing, extent and duration of the brief summer rains, and in good rainfall years the densely vegetated grasslands contrast sharply with the bare surfaces of FCs where there is little or no germination and thus recruitment of *Stipagrostis* seed [1]. This is in spite of the circle soils having a higher soil moisture content compared to those of the matrix [1,5,8,11]. If bare patches in the arid and extremely hot Namib Desert have higher soil temperatures than heavily grassed and shaded parts, this might negatively impact germination and seedling growth, contributing to the maintenance of the bare patches. Single exposure to very high temperatures or extended exposure to moderately high temperatures would potentially impact plant growth.

For brief periods seeds and seedlings would need to tolerate sustained daily soil surface temperatures of 45 °C and 47 °C on circles and the matrix respectively. During the hottest time of the day they would be exposed to soil surface temperatures of 56–60.5 °C (upper quartile) on the surface of circles and 59–62 °C (upper quartile) in the matrix, with the highest recorded soil temperatures reaching 63 °C on the circle and 68 °C in the matrix (Fig 1). In laboratory trials *S*. *ciliata* seeds germinated optimally between 35 °C to 37 °C–the temperature range where seedlings produced the highest number of roots and leaves, and the longest roots and leaves (Figs 2 and 3). This optimal temperature range for *S*. *ciliata* differs to that reported by Fakhfakh *et al* [32], who found that the highest germination percentages for this species occurred at 25 °C. This discrepancy could be explained either by Fakhfakh *et al* [32] using a constant experimental temperature approach, whereas in the current study seeds were exposed to the higher experimental temperatures for six hours each day instead of 24 hours, or by geographical variation in physiology of the species across its range. Both germination rate and growth of *S*. *ciliata* seedlings declined at temperatures above the optimum range of 35 °C to 37 °C, with virtually no germination, and little seedling growth occurring at 47 °C. Exposure to this temperature also resulted in the highest proportion of chlorosed leaves (Figs 2 and 3), a sign of heat stress [37]. Decreased germination rates, and suppression of root and shoot growth are typical of thermally stressed plants [38–40].

However, *Stipagrostis* seeds would only be exposed to such temperatures prior to being buried in the sand. The wind-dispersed seeds have a drilling awn that allows them to penetrate the soil [41], a trait which has been linked to adaptation to arid environments in some plant groups [42], allowing for some thermal buffering in deeper soils during germination and growth of the seedlings. Maximum soil temperatures recorded at 15 cm depth (the depth at which the seedling roots are situated–average *S*. *ciliata* root length is 10 cm after three weeks, pers. obs.) were 57.5 °C for matrix soils and 45.5 °C for circle soils. Exposure to these temperatures is likely to be brief, while at this depth seed and seedling development would subject to daily mean temperatures of 36.8 °C on FCs and 38.6 °C in the matrix throughout their summer growth period. This is 11–12 °C cooler than the mean daily temperatures on the soil surface. The daily field temperatures at 15 cm depth coincided with the optimum growing temperature range (35 °C to 37 °C) under experimental conditions.

Since soil temperatures at 15 cm below the bare disc matched the thermal optimum for *Stipagrostis* germination and seedling growth, this suggests that thermal properties of circle soils would not limit grass growth. Therefore, according to these findings, the hypothesis that high soil temperatures on the bare surface of FCs can exceed the thermal maxima of grass seedlings and thus lead to grass death can be rejected. Instead, circle soils were shown to be consistently 2 °C cooler than matrix soils, both on the surface and at 15 cm depth (Fig 1). Combined with the elevated soil moisture levels, the somewhat lower soil temperatures on the circles (when compared to those in the matrix) would apparently provide more favourable growth conditions when compared to matrix soils—opposite to what was initially hypothesised. Juergens [5] and Juergens et al. [3] likened FCs to water-rich oases, which also appear to offer more favourable thermal conditions for germination and seedling growth than the matrix. That FCs should provide an optimum growing condition in terms of water availability and soil temperature seems at odds with the plant competition hypothesis for circle formation [6,7] and the absence of vegetation on mature circles.

As the lack of shading on circle bare discs compared to the vegetated matrix did not result in hotter FC soils, another factor must play a role in keeping FCs relatively cooler. Soil moisture has been shown to lower and modify soil temperature [43,44]. Hence the most likely explanation for the lower soil temperatures on FCs is their elevated soil moisture content [1,5,8,11,45]. Prior to the onset of summer rains, FC’s may contain moisture levels 23 X greater than matrix soils. Water is an efficient temperature buffer due to its high heat capacity and role in evaporative cooling, and directly influences the temperature of the soil [46]. Since thermal, nutrient, and moisture conditions on circles appear optimal for seed germination and growth, there must be another factor that inhibits long-term grass recruitment on FC’s.

The Sand termite hypothesis suggests that FCs are a result of *P*. *allocerus* creating bare discs above their subterranean nest systems through destructive herbivory on grass roots and culms. This does not contradict the soil temperature observations made. In arid or semi-arid environments termites need to maintain constant high humidity conditions and specific temperature ranges within their nests to survive [47,48]. Many termites employ passive cooling and heating methods (occasionally active as in the case of metabolic heating) to maintain an optimal temperature range within their nests. This includes selective nest placement and alterations to the structure of the nest itself to offload or collect heat and moisture [49]. Whether the higher moisture and lower temperatures on FCs are an engineered adaptation of *P*. *allocerus* [5] is unclear, but regardless they likely provide a favourable homeostatic environment for the termites when compared to the significantly drier and hotter matrix soils.

## Conclusion

Although temperatures on the surface of both FC and matrix soils in NamibRand do reach above the thermal maximum for *S*. *ciliata*, maximum temperature at 15 cm depth was below the thermal maximum of 50 °C on FC soils. Therefore higher soil temperatures on FCs are unlikely to explain the maintenance of a bare patch through the prevention of grass seedling recruitment, and another mechanism must be responsible for the stark lack of vegetation on FCs. This study instead highlights a newly recorded soil characteristic of FCs in that they are consistently cooler than surrounding matrix soils, which with higher FC soil moisture should facilitate the growth of vegetation on FCs instead of preventing it.

## Supporting information

S1 Dataset Raw data for all in-situ temperature analyses and *S*. *ciliata* germination trials. (XLSX)

S1 Text Methods and results for pilot studies conducted prior to germination and seedling thermal experiments, investigating optimum thermal range, species seed viability, pre-wetting treatment and stratification treatment. (DOCX)
