# Supplementary material for: Do high soil temperatures on Namibian fairy circle discs explain the absence of vegetation?
Source: PLoS One. 2019 May 20;14(5):e0217153. doi: 10.1371/journal.pone.0217153 (PMC6527202; doi:10.1371/journal.pone.0217153)
Supplement: S1 Text — (DOCX) [file pone.0217153.s002.docx]

**Pilot study #1**

*S. ciliata* and *S. obtusa* seeds were exposed to temperatures of 30°C, 35°C, 40°C, 45°C and 50°C for 4-5 hours a day for 17 days after stratification. For the rest of the day, seeds were kept at room temperature. This was done to approximate thermal cycles experienced in nature. Prior to the experiment, all seeds were soaked in water for three hours before half were planted into pots and the rest stratified at 5°C for a further three days in wet conditions. For both species, four stratified and four non-stratified replicate pots were placed in each of the five water baths at the different temperatures, with 15 seeds planted upright just below the soil surface in each pot.

The first pilot experiment revealed that prior stratification of wetted seeds at 5 °C had no effect on germination success (χ² = 0.03, *P* = 0.869 for *S. ciliata* and χ² = 0.43, *P* = 0.511 for *S. obtusa*) (S2 Table 1). Germination success dropped at 45 °C, and none occurred at 50 °C for both species. These pilot studies informed the temperature range that was utilised in the two subsequent thermal tolerance trials of *S. ciliata* seed germination and transplanted seedlings.

S2 Table 1. Percentage (%) germination of *S. ciliata* and *S. obtusa* stratified and non-stratified seeds at 30 °C, 35 °C, 40 °C, 45 °C and 50 °C.

|  | **30°C** | **35°C** | **40°C** | **45°C** | **50°C** |
| --- | --- | --- | --- | --- | --- |
| ***S. ciliata* stratified** | 16.7 | 13.3 | 21.7 | 6.7 | 0 |
| ***S. ciliata* non-stratified** | 10 | 13.3 | 26.7 | 13.3 | 0 |
| ***S. obtusa* stratified** | 3.3 | 8.3 | 6.7 | 1.7 | 0 |
| ***S. obtusa* non-stratified** | 5 | 3.3 | 1.7 | 1.7 | 0 |

**Pilot study #2**

The effect of short-term exposure to 35°C, 37°C, 41°C, 44°C and 47°C on the germination success of both wetted and resting dry seeds (as would be in the dry season) for both species was tested. The minimum and maximum temperature from pilot study 1 (30°C and 50°C) were deemed redundant as both the optimum growth temperature and tolerance threshold occurred within these limits. Half of the seeds from each species were soaked for three days before planting and watered daily, while the remainder received no initial soaking or water for the first three days of temperature exposure. Stratification was not used for this pilot study as results from pilot study 1 indicated that stratification had no significant effect on the success of seed germination. Four pots with 30 seeds were used at each temperature, housing wetted and dry seeds of *S. ciliata* and *S. obtusa*. The seeds were subjected to the experimental temperatures for four hours on the first three days and allowed to rest at optimal growing temperatures for the remainder of the experiment, with the optimal growing temperatures determined from the previous pilot study (viz. 40°C for *S. ciliata* and 35°c for *S. obtusa*). The experiment was run for a total of 16 days, from the 19^th^ of October to the 3^rd^ of November 2011.

For the second pilot study, *S. obtusa* (and to a lesser extent *S. ciliata*) exposed to 35°C, 37°C, 41°C, 44°C and 47°C (temperature range modified based on previous pilot experiment) had greater germination success after being kept moist for three days prior to the experiment compared to those planted dry (Chi‑squared, χ² = 12.28, P = 0.001) (S2 Table 2). Overall *S. ciliata* had a significantly higher germination success than *S. obtusa* (χ² = 17.34, P = 0.000).

S2 Table 2. Percentage (%) of germinated wetted and dry *S. ciliata* and *S. obtusa* seeds at 35 °C, 38 °C, 41 °C, 44 °C and 47 °C.

|  | **35 °C** | **38 °C** | **41 °C** | **44 °C** | **47 °C** |
| --- | --- | --- | --- | --- | --- |
| ***S. ciliata* wet** | 33.3 | 13.3 | 26.7 | 20 | 26.7 |
| ***S. ciliata* dry** | 0 | 3.3 | 0 | 6.7 | 10 |
| ***S. obtusa* wet** | 6.7 | 0 | 0 | 0 | 3.3 |
| ***S. obtusa* dry** | 0 | 0 | 0 | 0 | 0 |
